# Bacterial gene loss as a mechanism for gain of antimicrobial resistance

**DOI:** 10.1016/j.mib.2012.07.008

**Published:** 2012-10

**Authors:** ME Török, N Chantratita, SJ Peacock

**Affiliations:** 1 Department of Medicine, University of Cambridge, Box 157, Addenbrooke's Hospital, Hills Road, Cambridge CB2 0QQ, United Kingdom; 2 Cambridge University Hospitals NHS Foundation Trust, Hills Road, Cambridge CB2 0QQ, United Kingdom; 3 Cambridge Health Protection Agency Microbiology and Public Health Laboratory, Box 236, Addenbrooke's Hospital, Hills Road, Cambridge CB2 0QQ, United Kingdom; 4 Department of Microbiology and Immunology, Faculty of Tropical Medicine, Mahidol University, Bangkok 10400, Thailand

## Abstract

Acquisition of exogenous DNA by pathogenic bacteria represents the basis for much of the acquired antimicrobial resistance in pathogenic bacteria. A more extreme mechanism to avoid the effect of an antibiotic is to delete the drug target, although this would be predicted to be rare since drug targets are often essential genes. Here, we review and discuss the description of a novel mechanism of resistance to the cephalosporin drug ceftazidime caused by loss of a penicillin-binding protein (PBP) in a Gram-negative bacillus (*Burkholderia pseudomallei*). This organism causes melioidosis across south-east Asia and northern Australia, and is usually treated with two or more weeks of ceftazidime followed by oral antibiotics for three to six months. Comparison of clinical isolates from six patients with melioidosis found initial ceftazidime-susceptible isolates and subsequent ceftazidime-resistant variants. The latter failed to grow on commonly used culture media, rendering these isolates difficult to detect in the diagnostic laboratory. Genomic analysis using pulsed-field gel electrophoresis and array based genomic hybridisation revealed a large-scale genomic deletion comprising 49 genes in the ceftazidime-resistant strains. Mutational analysis of wild-type *B. pseudomallei* demonstrated that ceftazidime resistance was due to deletion of a gene encoding a PBP 3 present within the region of genomic loss. This provides one explanation for ceftazidime treatment failure, and may be a frequent but undetected event in patients with melioidosis.

## Highlights

► We describe a novel ceftazidime resistance mechanism in *Burkholderia pseudomallei*. ► We analysed paired ceftazidime-susceptible and ceftazidime-resistant isolates from 6 patients. ► We found a large genomic deletion of 49 genes in the ceftazidime-resistant strains. ► We found that ceftazidime resistance was due to deletion of a PBP3 gene. ► This provides a possible explanation for ceftazidime treatment failure.

**Current Opinion in Microbiology** 2012, **15**:583–587

This review comes from a themed issue on **Antimicrobials**

Edited by **Didier Mazel** and **Shahriar Mobashery**

For a complete overview see the Issue and the Editorial

Available online 27th September 2012

1369-5274/$ – see front matter, © 2012 Elsevier Ltd. All rights reserved.

**http://dx.doi.org/10.1016/j.mib.2012.07.008**

## Introduction

Acquisition of exogenous DNA by pathogenic bacteria represents the basis for the inexorable increase in the prevalence of resistance to numerous classes of antimicrobial drugs in a wide range of bacterial species [1^•^]. Students of microbiology are taught the mechanisms of DNA acquisition at an early stage in their training, an understanding of which represents one of the most useful and durable set of principles for those who are interested in the biology of antibiotic resistance. The three mechanisms are transformation (direct uptake of exogenous DNA), conjugation (transfer of genetic material such as plasmids and transposons by direct cell-to-cell contact), and transduction (introduction of new genes via phage) [1^•^]. Introduction of new DNA may be associated with a fitness cost to the bacterium, but any disadvantage may be overcome in settings where the new phenotype provides a selective advantage. Healthcare settings are a case in point, where the emergence of a bacterial strain with a specific drug-resistant phenotype in response to antibiotic pressure may lead to clonal expansion and replacement of pre-existing strains. A good example is methicillin-resistance *Staphylococcus aureus* (MRSA), a resistant phenotype that results from the acquisition of a genetic element containing *mecA* encoding an altered penicillin-binding protein (PBP 2a) with lower affinity for all β-lactam antibiotics [2]. Much of the clinically relevant drug resistance arising in Gram-negative bacilli is due to gene acquisition, and includes the spread via mobile genetic elements of extended spectrum beta-lactamases [3^•^] and carbapenemases [4^•^]. A recent important example is the emergence and spread of Gram-negative bacteria positive for NDM-1 (New Delhi metallo-beta-lactamase) [5], which confers resistance to the carbapenem drugs, the drug class of choice for a range of situations where infection is potentially life threatening [6].

An alternative mechanism of antibiotic resistance is through mutation in existing gene(s) that encode the drug target. The development of resistance depends on the introduction of a mutation that leads to a fundamental change in the interaction between the drug and its bacterial target. For example, rifampicin, which is a broad spectrum antibiotic active against *Mycobacterium tuberculosis* and other bacterial pathogens, targets the DNA-dependent RNA polymerase β subunit, and resistance arises as a result of a mutation in the *rpoB* gene that encodes the rifampicin binding area [7]. A more extreme mechanism by which a bacterium could avoid the effect of an antibiotic is to delete the drug target altogether. This would be predicted to be extremely uncommon since drug targets are often essential genes, and gene loss would only be possible in the event that the function of the deleted gene could be performed by alternative genes or gene pathways. Here, we review and discuss the description of a novel mechanism of resistance to the cephalosporin drug ceftazidime based on loss of a PBP in a Gram-negative bacillus (*Burkholderia pseudomallei*).

## *B. pseudomallei* and melioidosis

*B. pseudomallei* is an environmental bacterium and the cause of melioidosis [8^••^]. This infection is most commonly seen in south-east Asia and northern Australia but has been reported worldwide, particularly in travellers returning from areas where meliodiosis is endemic. Infection can present with a wide spectrum of clinical features including septicaemia, pulmonary infection, intra-abdominal abscesses and disseminated infection [8^••^]. *B. pseudomallei* is intrinsically resistant to a range of antibiotics including gentamicin, streptomycin, rifampicin and many β-lactams. Reported resistance mechanisms include bacterial cell membrane impermeability [9], mutations in the antibiotic target site [10], enzymatic inactivation [11,12], and multi-drug efflux pumps [13,14]. The majority of *B. pseudomallei* isolates are susceptible to ceftazidime, trimethoprim-sulfamethoxazole, amoxicillin-clavulanate, imipenem and meropenem [15,16]. Antimicrobial therapy for melioidosis is required for three to six months to achieve cure, and is divided into an intravenous phase of ceftazidime or a carbapenem drug for two weeks (or longer if clinically indicated), followed by oral trimethoprim-sulfamethoxazole or amoxillin-clavulanate [17^•^]. The switch from parenteral to oral antimicrobial therapy is made once the patient shows clear evidence of clinical improvement, including an absence of fever for 48 hours and negative repeat blood culture taken at around one week after the onset of therapy. Prolonged parenteral therapy may be required for patients with disseminated infection, involvement of the central nervous system, bone or joint, and patients with deep-seated abscesses that cannot be drained. Despite the length of treatment, eradication of *B. pseudomallei* is notoriously difficult and high rates of clinical failure during the period of therapy and relapse from a persistent focus after antibiotics are stopped have been reported [18], although the basis for this is not understood. One possible explanation is the development of antibiotic resistance during therapy in a previously susceptible isolate (secondary resistance). No (primary or secondary) resistance to the carbapenem drugs has been reported in the published literature to date [15], while secondary resistance to ceftazidime has been reported in a very small number of cases; the mechanisms in some of these cases have been defined as mutations in the *penA* gene that encodes class A β-lactamase PenA and alters substrate specificity [19].

## Secondary resistance to ceftazidime in *B. pseudomallei*

The clinical narrative to this story starts in 2006, when a patient presented to a hospital in northeast Thailand with culture-confirmed melioidosis [20^••^]. *B. pseudomallei* was isolated from blood cultures, and was unremarkable in its growth characteristics and colonial appearance and was susceptible to ceftazidime. The patient was commenced on ceftazidime, but remained febrile after several weeks of therapy and underwent a splenectomy for large and persistent splenic abscesses. A subsequent blood culture taken on day 36 of ceftazidime treatment was culture negative on blood agar but grew pinpoint colonies after 48 hours of incubation on a solid medium called Ashdown agar, a selective medium used specifically for the culture of *B. pseudomallei* [21^•^]. The colonial morphology was unusual for *B. pseudomallei* (which normally produces characteristic ‘cornflower head’ colonies), and Gram stain revealed Gram-negative filaments. Identification using routine biochemical tests was unsuccessful because of very poor bacterial growth, but a monoclonal antibody-based latex agglutination test to *B. pseudomallei* exopolysaccharide was positive [22]. Antimicrobial therapy was changed to oral trimethoprim-sulfamethoxazole, the patient became afebrile and was discharged from hospital on day 53. Two similar cases occurred in 2007 and examination of laboratory records identified three further cases (six cases in total), all of whom had been treated with prolonged ceftazidime therapy (median 26.5 days, range 18–36 days), had failed to respond to therapy, and grew a bacterial variant that was similar to the first case. The highly atypical morphological appearance on solid agar would almost certainly result in most such cultures being considered as contaminants of no clinical significance.

**Figure 1:**
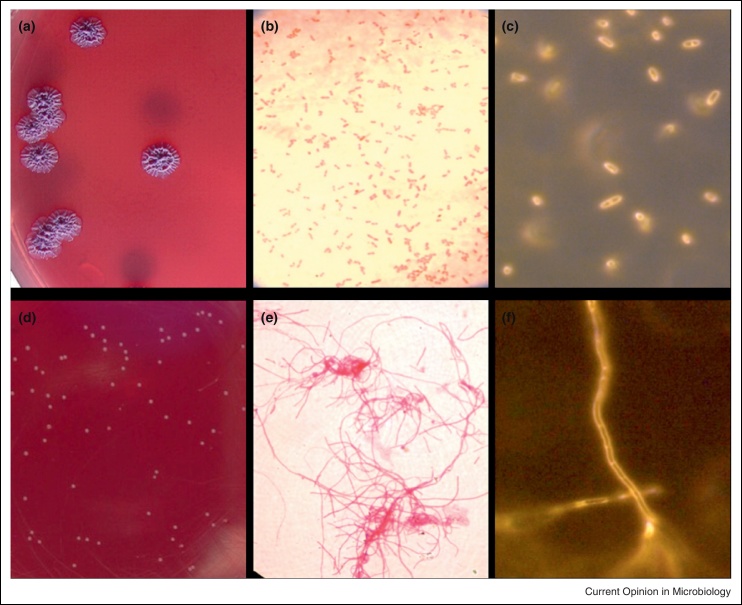
Comparison of the appearance of an initial ceftazidime-susceptible *B. pseudomallei* strain 415a and the ceftazidime-resistant variant strain 415e isolated from the same patient after prolonged ceftazidime therapy. Colony morphology **(a and d)**, Gram stain and light microscopy **(b and e)**, and unstained appearance by real-time microscopy **(c and f)** of initial (a to c) and variant strain (d to f). Colony morphology was observed after spread plating on Ashdown agar and incubation for 4 days at 37 °C in air. Gram stain was observed through a 40× objective. Real-time microscopy was performed using a real time microscope (RTM-3) at 1000× magnification. Reproduced with permission from Chantratita *et al.* [20^••^].

A series of simple growth experiments were performed on pairs of isolates from the six cases (first isolate to be cultured on admission and subsequent variant strain), to test whether these grew on commonly used bacteriological media. The admission isolates had typical growth characteristics and colonial morphology on a range of solid media including blood agar, Columbia agar, Mueller-Hinton agar, tryptone soya agar, *Burkholderia cepacia* agar, Luria–Bertani agar and Ashdown agar (Figure 1a). These also grew in commercial blood culture bottles, tryptone soya broth and Mueller-Hinton broth. In contrast, despite prolonged incubation for seven days at 37 °C, the variant isolates failed to grow on any of the media apart from Ashdown agar, on which pinpoint colonies were seen after 48 hours’ incubation (Figure 1d). The six variant strains also failed to grow in the commercial blood culture bottles or tryptone soya broth. This finding has major implications for clinical care, since it is highly likely that culture of samples containing variant *B. pseudomallei* would be falsely negative. Gram stain of the admission isolates was typical for the species (Figure 1b), but Gram stain of all of the variant isolates showed Gram-negative filaments (Figure 1e). In the diagnostic laboratory, this appearance would mean that *B. pseudomallei* would not be considered. Real-time microscopy (RTM-3) which allows visualization of live bacteria in the absence of stains (or fixatives) of the six initial strains demonstrated motile bacilli (Figure 1c), whereas the six variant strains were nonmotile filaments with an appearance consistent with the presence of septa in the absence of cell division (Figure 1f).

Antimicrobial susceptibility testing was performed on the six isolate pairs; this confirmed that the admission isolates were susceptible to ceftazidime but that the variants were highly resistant (minimum inhibitory concentration [MIC] > 256 μg/ml). MICs for other antimicrobial classes demonstrated that within-pair MICs were comparable for the six isolate pairs (including amoxillin-clavulanate), indicating that the defect appeared to be specific to ceftazidime. Resistance most likely arose *in vivo* during ceftazidime therapy, a suggestion supported by genotyping data which showed that isolate pairs from the same patient were the same genotype (as defined by multilocus sequence typing). Each patient was infected with a different genotype, however, suggesting that the ceftazidime-resistant variant had arisen independently in several lineages. Two of the six patients died, which is comparable to the crude mortality rate from melioidosis in the same hospital setting. This suggests that the variants remained virulent in the human host despite the obvious growth defects under *in vitro* conditions.

## Gain of resistance through gene loss

Sequencing of the *penA* gene of the admission and variant isolates failed to identify any *de novo* genetic changes in the *penA* gene, suggesting a novel resistance mechanism. Comparison of the banding pattern produced by pulsed-field gel electrophoresis (PFGE) [23] between each strain pair showed a loss of a 150 kb band in four of the six variant strains, suggesting a large genomic deletion. This was further investigated by array based comparative genomic hybridisation (aCGH) [24]. *B. pseudomallei* has two chromosomes, and aCGH demonstrated a genomic deletion in chromosome 2 in all six variant strains. These ranged in size from 145 kb to 309 kb, with a minimal common region of genomic loss of 71 kb comprising 49 genes. PCR and sequencing in the region of the putative deletion were used to confirm the deletion. Analysis of the flanking sequences did not identify any distinct motifs associated with breakpoints, suggesting that the most likely mechanism was random recombination in the presence of ceftazidime.

The common deleted region included three genes that were potential candidates for the resistant phenotype — two encoded penicillin-binding proteins of the PBP 3 family, and the third encoded a putative d-alanyl-d-alanine carboxypeptidase that belongs to the PBP 5/6 family. Mutants were made in each gene using a laboratory strain of *B. pseudomallei*, which implicated one of the PBP 3 genes (BPSS1219). After a series of complex molecular steps to circumvent what appeared to be a lethal mutation when BPSS1219 was rendered detective, this gene was shown to be associated with the growth detect, filamentation, and resistance to ceftazidime [20^••^]. This finding is compatible with several lines of evidence in the literature. In other Gram-negative bacilli including *Escherichia coli* and *Pseudomonas aeruginosa*, ceftazidime owes its antibacterial activity to a high affinity for PBP 3 [25]. In addition, inactivation of PBP 3 in *E. coli* results in inhibition of cell division and growth into long filaments [26,27]. The growth defect of the variant *B. pseudomallei* with very slow growth on Ashdown agar but no other media may be related to osmotic effects and bacterial lysis, with growth on Ashdown being supported by the presence of 4% glycerol.

## Gene loss and gene gain by *B. pseudomallei*

The *B. pseudomallei* genome is highly dynamic, with around 15% of the genome being variably present across isolates [28–30]. The variable region includes multiple genomic islands containing DNA acquired from other bacteria. There is also existing evidence for genetic divergence of *B. pseudomallei* during human infection, which was demonstrated by genotyping multiple colonies from several tissue sites of patients with acute melioidosis [31]. Natural, large-scale deletion of genomic material in *B. pseudomallei* has been reported once before [32]. *B. pseudomallei* is intrinsically resistant to gentamicin, and deletion of a region of >130 kb including the *amrAB-oprA* operon is the basis for gentamicin-susceptible strains (a phenotype which occurs in 1 in 1000 clinical isolates) which remain virulent in patients.

## Concluding comments

Gene deletion is an extreme and rare mechanism of gain of resistance to antimicrobial drugs. A fascinating and clinically relevant twist to the story recounted here is that the resistant *B. pseudomallei* variants were rendered almost undetectable in the diagnostic microbiology laboratory. It remains to be seen as to what proportion of patients who fail ceftazidime therapy for melioidosis fall into the category of having such a variant.

## References and recommended reading

Papers of particular interest, published within the period of review, have been highlighted as:

• of special interest
•• of outstanding interest
